# External review and validation of the Swedish national inpatient register

**DOI:** 10.1186/1471-2458-11-450

**Published:** 2011-06-09

**Authors:** Jonas F Ludvigsson, Eva Andersson, Anders Ekbom, Maria Feychting, Jeong-Lim Kim, Christina Reuterwall, Mona Heurgren, Petra Otterblad Olausson

**Affiliations:** 1 Department of Paediatrics, Örebro University Hospital, Sweden; 2 Clinical Epidemiology Unit, Department of Medicine, Karolinska University Hospital, Karolinska Institutet, Sweden; 3 Section of Occupational and Environmental Medicine, University of Gothenburg, Sweden; 4 Institute of Environmental Medicine, Karolinska Institutet, Sweden; 5 Research Unit, Jämtland County, Sweden; 6 Department of Public Health and Clinical Medicine, Umeå University, Sweden; 7 Department of Statistics and Analyses, National Board of Health and Welfare, Stockholm, Sweden

**Keywords:** Classification of diseases, disease, epidemiology, morbidity, register

## Abstract

**Background:**

The Swedish National Inpatient Register (IPR), also called the Hospital Discharge Register, is a principal source of data for numerous research projects. The IPR is part of the National Patient Register. The Swedish IPR was launched in 1964 (psychiatric diagnoses from 1973) but complete coverage did not begin until 1987. Currently, more than 99% of all somatic (including surgery) and psychiatric hospital discharges are registered in the IPR. A previous validation of the IPR by the National Board of Health and Welfare showed that 85-95% of all diagnoses in the IPR are valid. The current paper describes the history, structure, coverage and quality of the Swedish IPR.

**Methods and results:**

In January 2010, we searched the medical databases, Medline and HighWire, using the search algorithm "validat* (inpatient or hospital discharge) Sweden". We also contacted 218 members of the Swedish Society of Epidemiology and an additional 201 medical researchers to identify papers that had validated the IPR. In total, 132 papers were reviewed. The positive predictive value (PPV) was found to differ between diagnoses in the IPR, but is generally 85-95%.

**Conclusions:**

In conclusion, the validity of the Swedish IPR is high for many but not all diagnoses. The long follow-up makes the register particularly suitable for large-scale population-based research, but for certain research areas the use of other health registers, such as the Swedish Cancer Register, may be more suitable.

## Background

**Figure 1:**
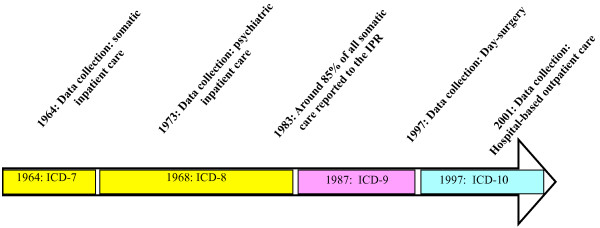
**Timeline of the Swedish Inpatient Register**. Years inside the arrow indicate the first year when an ICD classification was in use. ICD-10 was introduced in 1997, with the exception of the county of Skåne where ICD-9 was still in use throughout 1997. The one-year delay in introducing ICD-10 in Skåne has some implications when identifying patients with a certain disease/disorder in this county because about 8-9% of the Swedish population live in Skåne.

The Swedish National Inpatient Register (IPR; Swedish: slutenvårdsregistret), also called the Hospital Discharge Register, was established in 1964 (Figure 1). The IPR has complete national coverage since 1987. The IPR is part of the National Patient Register (Swedish: patientregistret). Currently, more than 99% of all somatic and psychiatric hospital discharges are registered in the IPR. Diagnoses in the IPR are coded according to the Swedish international classification of disease (ICD) system, first introduced in 1964 (adapted from the WHO ICD classification system) (Figure 1). A history of the Swedish and Nordic ICD system has been published elsewhere [1]. It is mandatory for all physicians, private and publicly funded, to deliver data to the IPR (except for visits in primary care). A detailed description of the regulations relevant to the IPR has been given in the Appendix (Additional file 1).

### History and coverage of the IPR

**Figure 2:**
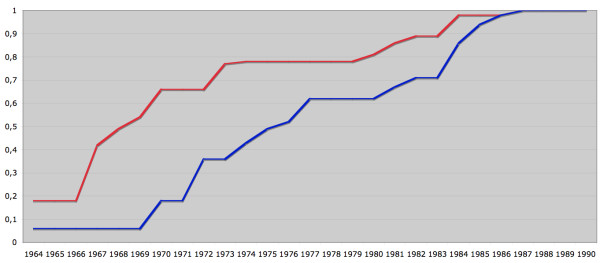
**Somatic care: coverage of the Swedish population**. Red = Proportion of the Swedish population living in counties that had started to report somatic hospital discharges to the Swedish Inpatient Register. Blue = Proportion of the Swedish population living in counties where all somatic hospital discharges were reported to the Swedish Inpatient Register (1964: 6%; 1972: 36%; 1982: 71%; 1984: 86%). In 1976, for the first time more than 50% of the Swedish population were covered. Complete coverage (100%) was attained in 1987. County population data obtained from the government agency Statistics Sweden (Appendix).

**Figure 3:**
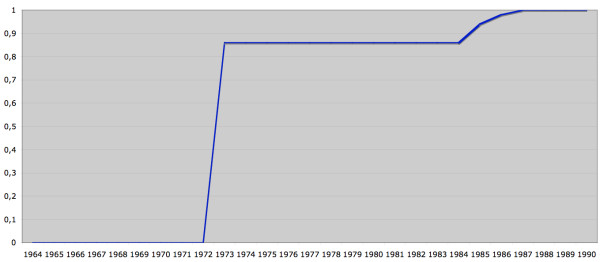
**Psychiatric care: coverage of the Swedish population**. Blue = Proportion of the Swedish population living in counties where all psychiatric hospital discharges were reported to the Swedish Inpatient Register (1973: 86%; 1985: 94%; 1986: 98%). All counties in Sweden started to record psychiatric care in 1973. (Actually, psychiatric diagnoses were recorded before 1973 but then removed until 1973 - see text). County population data obtained from the government agency Statistics Sweden (Appendix).

The IPR was founded in 1964 when the NBHW (National Board of Health and Welfare; Swedish: Socialstyrelsen) began collecting data on somatic inpatient care in six Swedish counties (roughly the Uppsala region)(Figure 2, red line)[2] (for the population statistics underlying Figures 2 and 3, please see Additional file 2). In fact, the NBHW started to collect data on psychiatric care in 1962 but when the IPR was reconstructed in the 1990s, all psychiatric data originating before 1973 were removed (Figure 3). Beginning in about 1970, data collection for the IPR went from a pilot project to an all-inclusive effort to cover the entire country. In 1983, approximately 85% of all somatic care and almost all psychiatric care were reported to the NBHW [2]. In 1984, the NBHW asked permission from the National Data Inspection Board to link individual data to the personal identity number (PIN) (Swedish: personnummer) [3] of each individual. Although granted permission, the NBHW postponed the introduction of a PIN-based register because the Swedish attorney general objected to the use of the PIN in the IPR. Only in 1993 did the Swedish government declare that the IPR should use the PIN as the unique identifier in all hospital discharges. After 1993, all counties have collaborated on reconstructing earlier hospital discharges linked to the PIN for the years 1984-91. This linkage was possible for all but three counties: two counties were unable to reconstruct data for the year 1985 while the third did not enter the IPR until 1987.

**Figure 4:**
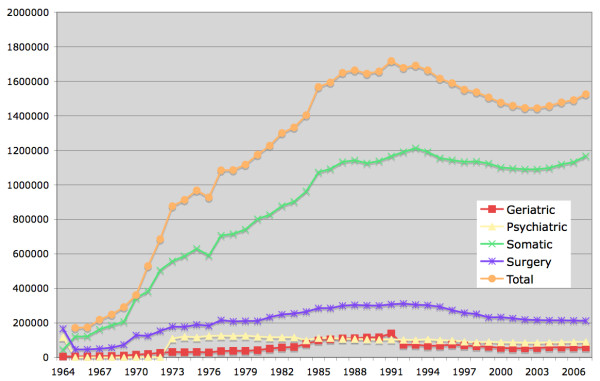
**Number of hospital discharges from 1964-2007**[2]. Surgery = General surgery.

Each year, there are about 1.5 million hospital discharges in the IPR (Figure 4), with the majority of these taking place in somatic care. From 1997 and onwards, surgical day care procedures are reported to the NBHW, and since 2001, counties are obliged to report hospital-based outpatient physician visits. However, primary health care data are still not reported on a national level to the NBHW. Whereas coverage of the IPR is currently almost 100%; coverage of hospital-based *outpatient *care is considerably lower (about 80%)[2]. In the outpatient register, data from private caregivers are missing (coverage of data from public caregivers in outpatient care is almost 100%). The number of hospitals reporting to the IPR increased rapidly in the 1970s. In the 1960s, 20 hospitals and roughly 80 nursing homes reported to the IPR [2]. In the 1980s, the number of units reporting to the IPR had increased to 580. Because of organizational changes, the number of reporting units has since declined.

### IPR variables

**Figure 5:**
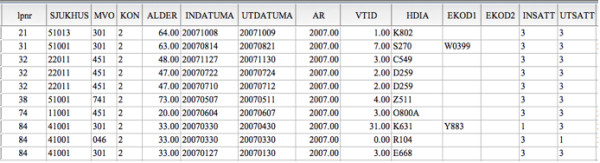
**A sample of variables from the Swedish Inpatient Register (as seen with the statistics programme SPSS)**. Each hospital discharge is listed on a row. This means that an individual may occupy several rows in the IPR (first, second, third hospital discharge, etc.). The variable lpnr (or lopnr) is constructed when the dataset is delivered to the researcher, and serves as unique serial number. In the original IPR dataset, each discharge is linked to a unique Personal Identity Number (PIN)[3]. Please note that the order of the variables above may differ from that in the original IPR dataset.

IPR variables can be divided into four categories: patient-related data, data about the caregiver, administrative data and medical data (Table 1). Figure 5 displays a typical dataset from the IPR as delivered to researchers.

**Table 1:** Variables in the Swedish IPR

Variable	Description
**Patient-related data**	
Personal Identity Number (PIN)	Combination of date of birth, three-digit birth number and a check digit [3]. Personal identity number shall be reported for all admissions/discharges, except for induced abortions where it is not registered for legal reasons
Sex	1 = male; 2 = female
Age	Age in years at discharge. In individuals with missing birth dates, the difference between year of discharge and birth year is used to calculate age.
County	The county where the patient has his/her permanent residence (this is not necessarily the county where the patient is admitted).
Municipality and parish	Usually consists of six digits, where positions 1-2 indicate county, 3-4 municipality and 5-6 parish. Individuals living outside Sweden are assigned the value "99". Missing data have been replaced by data from Statistics Sweden.
**Data about caregiver (hospital/department)**	
Hospital	Each hospital in Sweden has a unique 5-digit code assigned by the National Board of Health and Welfare (NBHW)(e.g., Lund University Hospital has code 41001).
Type of department	Each type of department or health centre has a unique code assigned by the NBHW (e.g. ophthalmology departments have code 511)
**Administrative data**	
Admission date	Year-month-day
Discharge date	Year-month-day
Duration of admission	Number of days at hospital. Patients discharged on the day of admission are assigned the value "1".
Elective health care	1 = Yes, 2 = No
Mode of admission	1 = from other hospital/department, 2 = from special living (e.g., home for disabled people, or geriatric care), 3 = other (i.e. from home)
Mode of discharge	1 = to other hospital/department, 2 = to special living (e.g., home for disabled people or geriatric care), 3 = other (i.e. discharged to home), 4 = deceased.
**Medical data**	
Diagnoses	In 1964-1996, the IPR permitted up to 6 diagnoses per discharge. Between 1997-2009 8 diagnoses could be recorded (one of them being the primary diagnosis).
Primary and additional diagnoses	The primary diagnosis or "main condition" should be the condition diagnosed at the end of the episode of health care responsible for the patient's need for treatment or investigation. The additional (secondary or contributory diagnoses/conditions) may or may not contribute to the primary diagnosis. They may be co-morbidities and/or complications. Since 2010 the number of possible additional diagnoses per case is unlimited (however, the NBHW will generally only deliver the first 7 additional diagnoses to researchers who request data from the IPR).
External cause of injury or poisoning (E-code) - or "Chapter XX codes".	Until 1997, only one E-code could be recorded per discharge; from 1998, numerous "E-codes" may be recorded. With the introduction of ICD-10 in 1997, E-codes should be referred to as "Chapter XX-codes". (In ICD-10, E00-E99 codes represent metabolic conditions).
Procedures	In 1964 the Swedish NBHW introduced a national classification of procedures based on an American classification of surgical procedures. It had four digit-codes (e.g. appendectomy 4510). Since 1997, a Swedish version of the NOMESCO Classification of Surgical Procedures is in use. This classification is based on five-character alpha-numeric codes (e.g. JEA01 for appendectomy). Current procedures are listed in the Swedish Classification of surgical and medical procedures (Swedish: "KVÅ" - klassifikation av vårdåtgärder)(issued by the NBHW). Between 1964 and 1996, up to 6 operations/surgical procedures could be listed per discharge. From 1997, up to 12 operations/surgical procedures could be listed per discharge. In the future it will be possible to record more than 12 diagnoses per discharge. Since 2007, all performed procedures are mandatory to record, including medical procedures. The surgeon may also (voluntarily) report date of operation and type of anaesthesia and drugs used according to the ATC list.
Psychiatric care	0 = voluntary care, 1-4: compulsory psychiatric inpatient care (under different conditions or according to certain laws). If a patient has been treated according to categories 1, 2, 3 or 4, the condition prevailing most of the time shall be reported. Compulsory care can be further divided into "forensic" and "civil", depending on the reasons for compulsory care.

IPR = Inpatient Register. NBHW = National Board of Health and Welfare.

Since January 2009, the NBHW collects additional data on compulsory psychiatric care (psychiatric care under certain laws) in addition to the IPR. The data are collected three times per year.

In older versions of the IPR, the variable "Billing forms (between counties)" was also included.

The basic unit of the IPR is not the patient but the admission/discharge. Individual patients can be identified by their unique PIN.

#### Personal identity number (PIN)

Each hospital discharge is keyed to an individual's PIN [3] (Table 1). Overall (1964-2008), the PIN is found missing in 2.9% of all hospital discharges.

#### Primary diagnosis

Overall, a primary diagnosis is listed in 99% of all hospital discharges. The highest rate of missing data occurred in 1968 (4.6%), which may be due to the change from ICD-7 to ICD-8 that occurred in that year. After 2000, missing primary diagnoses have been consistently more common in psychiatric care than in somatic care (5.7-9.4% in psychiatric care vs. 0.5-0.9% in somatic care). Since the start of the IPR, primary diagnoses are missing in 0.8% of somatic care, 2.4% of geriatric care, 3.1% of psychiatric care and 0.5% of general surgery.

The proportion of patients without a primary diagnosis does not differ by hospital type (university hospitals 1.4%, county hospitals 0.7%, small local hospitals 0.8%) but is slightly higher in nursing homes (3.1%).

#### Injuries and poisoning: external cause

**Figure 6:**
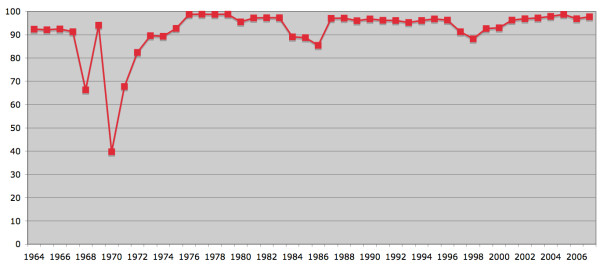
**Percentage of hospital discharges for injury and poisoning with reported external cause**[2] .

All hospital admissions for injury or poisoning must be coded by an E code indicating the cause of the injury/poisoning (Figure 6).

#### Mode of admission and discharge

The variables "mode of admission" and "mode of discharge" describe where the patient stays before and after admission, respectively (Table 1). These variables have generally been recorded in more than 95% of all hospital admissions (with the exception of the year 1979 and in single counties in 1997-2000).

#### Alternative registers

Even though the IPR contains important information on a wide spectrum of diagnoses, it is sometimes preferable to use other Swedish health registers, such as the *Swedish Cancer Register*)[4], the *Cause of Death Register*[5] and the Swedish *Medical Birth Register*[6]. There are also a large number of *Swedish National Quality Registers *(n = 89 in 2011)(http://www.kvalitetsregister.se, accessed April 19, 2011).

### Earlier assessment of the IPR

The NBHW has previously examined the quality of the IPR on three separate occasions (one published study with data collection in 1986 (899 patients, patient chart validation)[7], one unpublished study with data collection in 1990 (n = 875, patient chart validation)[2,8] and one comparison between the IPR and the National Quality Registers in 2009. The two patient chart studies focused on three types of diagnostic coding error detected in medical records.

*1. Diagnostic errors*, i.e. the patient received an incorrect diagnosis (the patient receives an ICD code that is not related to his or her actual main complaint). Diagnostic errors were more common in internal medicine records (especially in the 1986 study [7]) than in records from gynaecology departments, and slightly more common in older than in younger patients [2].

*2. Translation errors*, i.e. the ICD code in the *IPR *is different from the code actually listed in the *patient chart*. This type of error was detected in less than 1% of all medical records.

*3. Coding errors*, i.e. the faulty ICD code accompanies an otherwise correct diagnosis. Such coding errors occurred in 5.9% of hospital discharges in 1986 and in 8.3% in 1990.

In the 1990 validation, the risk of an incorrect primary diagnosis correlated with the number of secondary diagnoses [8]. The overall proportion of incorrect diagnoses at the ICD code 3-digit/character level (e.g., ICD-9: 571 "chronic liver disease and liver cirrhosis") was 13% in 1986 and 12% in 1990; at the four-digit level (e.g., ICD-9: 571E "chronic hepatitis"), it was 15% in 1986 and 14% in 1990 (B. Smedby, personal communication, Jan 30, 2010).

The comparison between the IPR and the National Quality Registers found that the IPR has high sensitivity for most surgical procedures (Table 2)[9], whereas sensitivity varied between 76.4% and 96.0% for three diseases not requiring surgery (multiple sclerosis, incident stroke and prostate cancer)(Table 2).

**Table 2:** Comparison between Swedish Quality Registers and the National Patient Register [9]

Disease/ Procedure	Quality Register	Total number of cases	Matching between the Quality Register and the Patient Register (%)	Proportion of all cases identified through the Patient Register (%)
Hernia surgery*	Swedish Hernia Register	17,707	69.9	92.5
Cholecystectomy*	Swedish Register of Gallstone Surgery and ERCP	12,472	79.9	96.4
ERCP*		7,458	54.5	71.2
Multiple Sclerosis*	Swedish Multiple Sclerosis Register	13,503	52.9	76.4
Knee arthroplasty	The Swedish Knee Arthroplasty Register	11,122	90.1	93.6
Hip arthroplasty	The Swedish Hip Arthroplasty Register	14,757	91.0	93.4
Hip fracture	The Swedish Hip Fracture Register	15,920	64.0	95.3
Surgery on the abdominal aorta*	Swedvasc	1,784	77.4	90.6
Infrainguinal bypass surgery		979	72.4	81.9
Carotid artery surgery		1,584	81.6	95.2
First stroke	Swedish Stroke Register	22,202	79.4	96.0
Heart surgery	Swedish Heart Surgery Register#	13,440	95.1	97.6
Cataract surgery*	National Cataract Register	75,050	75.2	78.6
Prostate cancer*	National Prostate Cancer Register	3,985	62.0	82.8

ERCP = endoscopic retrograde cholangiopancreatography

* Quality register compared with the Patient Register (IPR *and *hospital-based outpatient data). In the other comparisons the Quality Register was compared only with the IPR.

# Currently part of the Register *Swedeheart*.

### Use of the IPR

Systematic collection of medical data is essential for modern health care because such data are used to plan, evaluate and fund health care. Through the IPR, administrators, health care personnel and researchers are able to (a) evaluate the incidence and prevalence of diseases [10], (b) examine the effects and consequences of interventions (e.g., surgery [11]), including quality of care and (c) establish cohorts of patients with a certain disease [12] or condition.

The primary purpose of this paper was to review and validate the IPR. A second objective was to describe its potential use in population-based epidemiological research.

## Methods

Sorensen et al suggest that administrative databases could be evaluated in three ways [13]:

(a) Through comparison with other independent reference sources

(b) Through patient chart reviews (medical records)

(c) By comparing the total number of cases in different databases

The majority of the evaluations in this paper were based on (b), i.e. patient chart reviews.

### Assessment by the current study

**Figure 7:**
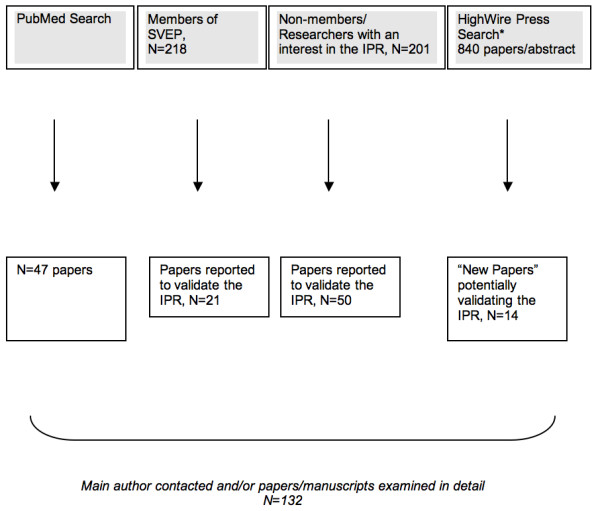
**Collection of validation studies**. In both the PubMed and HighWire Press search, we used the following search algorithm to identify relevant papers: validat* (inpatient or hospital discharge) Sweden. Databases were searched from the start of the databases until January 2010. *In the HighWire Press literature search, JFL manually screened all titles, authors, keywords and, when available, abstracts for the 840 hits. If a validation of the inpatient register could not be ruled out, the corresponding author was contacted. A number of publications could then be excluded; 14 "new papers" remained that had not previously been identified.

In January 2010, we began identifying papers that might concern the validity of the IPR (Figure 7) using database searches in PubMed and HighWire. We used the following search algorithm: "validat* (inpatient or hospital discharge) Sweden". We also contacted 218 members of the Swedish Society of Epidemiology and another 201 researchers with experience in register-based research. Altogether, we identified 132 papers, all of which were subsequently examined in detail. Tables 3 and 4 list papers that validated the IPR.

**Table 3:** Validation of diagnoses in the Swedish Inpatient Register by Positive Predictive Values (PPVs)

Diagnosis	Correct diagnosis in the IPR (%) = positive predictive value (PPV)	Comment	Main Author	PubMed ID	Year	Gold Standard (reference)
**Cardiovascular disease**						
Myocardial infarction	36/36 (100)	Only primary diagnoses were evaluated. NPV = 861/864 (99.7)	Nilsson [7]	8114596	1994	MR
Myocardial infarction	2053/2101 (98)	Patients aged 45 - 70 years in 1992 - 1994.	Linnersjö[34]	11121592	2000	MR
Angina Pectoris	18/19 (95)	Only primary diagnoses were evaluated. NPV = 879/881 (99.8)	Nilsson [7]	8114596	1994	MR
Heart failure (HF)	259/317 (81.7)	Definition of HF proposed by the European Society of Cardiology [35]. Restricting the diagnosis HF to the primary diagnosis, then 133/140 (95.0%) were correct.	Ingelsson [36]	15916919	2005	ULSAM
Heart failure	15/17 (88)	Only primary diagnoses were evaluated. NPV = 876/883 (99.2)	Nilsson [7]	8114596	1994	MR
Atrial fibrillation (using ICD-codes for atrial fibrillation or atrial flutter)	97/100 (97)	95 individuals had ECGs consistent with atrial fibrillation. Two patients had no ECGs available but were regarded as having atrial fibrillation on the basis of medical records.	Smith [37]	19936945	2009	MR + Electrocardiograms (ECG)
Non-fatal strokes	3492/5101 (68.5)	Patients aged 25-74 years in 1985-89. Criteria for acute stroke in this study were based on WHO criteria.	Stegmayr [38]	1291884	1992	MONICA-population based stroke register
Stroke/Transient ischemic Attack	207/210 (98.6)	ULSAM	Wiberg		(PC, Feb 12)	ULSAM
Gestational hypertension	108/111 (97.3)		Zetterström		(PC, March 3)	MR
Gestational hypertension	97/115 (84.3)		Ros [39]	9620050	1998	MR
Vascular interventions (for lower limb ischemia)	545/546 (99.8)		Hultgren [40]	11170873	2001	MR
**Autoimmune/immune-mediated diseases**						
Rheumatoid arthritis (RA)	489/510 (95.9)	Malmö, Sweden, 1990-1994. According to the RA criteria of the American College of Rheumatology [41].	Turesson [42]	10461483	1999	MR
Rheumatoid arthritis	Without lymphoma: 440/505 (87.1) With lymphoma: 386/413 (93.5)	In 40 of the 386 cases with lymphoma, medical records did not include enough information to evaluate whether the RA criteria of the American College of Rheumatology were fulfilled but all available information supported the diagnosis of RA	Baecklund [22]	16508929	2006	MR
Wegener's granulomatosis	68/78 (87)	American College of Rheumatology diagnostic criteria [43]	Knight [12]	12115591	2002	MR
Celiac disease	66/77 (86) of patients with later lymphoma	Only in 8 patients could celiac disease be ruled out. In 3 patients, the chart reviews were consistent with possible celiac disease.	Ekström-Smedby [44]	15591504	2005	MR
Primary adrenocortical insufficiency	105/133 (78.9)		Bensing [45]	18727712	2008	Patients tested positive for 21-OH autoantibodies
Diabetes mellitus (type 1 and 2)*	22/28 (79)	Only primary diagnoses were evaluated. NPV = 872/872 (100)	Nilsson [7]	8114596	1994	MR
Both diabetes (type 1 or 2) and foot ulcer*	235/236 (99.6) admissions were correct with regards to ICD-coding	117 patients with deep foot infections and type 1 or 2 diabetes, who had been referred to a multidisciplinary foot-care team at Lund University Hospital.	Ragnarson-Tennvall [46]	11123504	2000	MR
Inflammatory bowel disease (IBD)	4778/6440 (74)	1965-1983. Due to the ICD-classification used at this time, ulcerative colitis could not be distinguished from Crohn's disease, and therefore "overall IBD" was evaluated	Ekbom [47]	1985033	1991	MR + histopathological reviews
**Psychiatric disease and neurology**						
Schizophrenia	94/100 (94.9)	Review of medical records with structured diagnostic interviews	Ekholm [48]	16316898	2005	MR
Schizophrenia	78/91 (85.7)	Individuals born 1973-77. DSM-IV criteria for schizophrenia syndrome	Dalman [49]	12395142	2002	MR
Schizophrenia	106/111 (95.5)	Review of 121 consecutive cases in one city using a structured DSM-IV checklist, 111 records obtained	Hultman [50]	16863597	2006	MR
Schizophrenia, schizophreniform disorder or schizoaffective disorder	94/168 (56)	Primary diagnoses that fulfilled DSM-IV criteria (OPCRIT algorithm)	Reutfors [51]	-	2009	MR
Schizophrenia †	78/104 (75.0) to 85/104 (81.7)	104 patients discharged in 1971 with a diagnosis of schizophrenia. DSMIII-criteria were used for this validation. From Stockholm County IPR. A strict review found a PPV of 75%. This figure increased to 85% when some lack of information was accepted (less strict criteria).	Kristjansson [52]	-	1987	MR
Schizophrenia	(Kappa = 0.37 correlating to an agreement rate of 68%)	All individuals with a diagnosis of schizophrenia in the IPR and who had an inpatient forensic psychiatric assessment using a national register of all such evaluations from 1988-2000 (n = 1638).	Fazel [25]	19454640	2009	Forensic psychiatric assessment
Schizophrenia	34/44 (77%)		Bergman [53]	-	1999	Four-week- inpatient assessment in forensic psychiatry department
Alzheimer	54/75 (72)		Jin [54]	15326258	2004	Clinical work-up following phone interview
Personality disorders	37/40 (92)	Random sample of 40 individuals out of 401 violent offenders with personality disorders from a longitudinal study	Grann [55]	-	1998	MR
Personality disorders	30/55 (55%)		Bergman [53]	-	1999	Four-week- inpatient assessment in forensic psychiatry department
Guillain-Barré Syndrome (GBS)	69/83 (83)	83% of patients fulfilled the National Institute of Neurological and Communicative Disorders and Stroke criteria for GBS [56]. PPV of first primary diagnosis with GBS was 84%; and for secondary diagnosis 75%.	Jiang [57]	7785420	1995	MR
Herpes simplex encephalit	223/638 (35.0)	1990-2001	Hjalmarsson [58]	17806053	2007	Laboratory data (positive finding of HSV-1)
**Trauma and fractures**						
Brain concussion	18/18 (100)	Only primary diagnoses were evaluated. NPV = 880/882 (99.8)	Nilsson [7]	8114596	1994	MR
Hip fracture	21/22 (95)	Only primary diagnoses were evaluated. NPV = 877/878 (99.9)	Nilsson [7]	8114596	1994	MR
Hip fracture	2556/2597 (98.4)	Cases derived both from IPR and operation registers.	Michaelsson [59]	9632404	1998	MR
Injuries	1299/1370 (94.8)	Injury code was correct at 3-digit-level.	Gedeborg	-	(PC, Feb 3)	MR
**Surgical procedures (and related medical conditions)**						
Major amputations (leg and arm)	610/624 (97.8)	Unique number of amputations was 610 in 624 patients.	Malmstedt		(PC, Feb 24)	MR
Appendicitis†	1661/1840 (90.3)	Performed in the Jönköping county. Incidental appendectomies were excluded. The IPR overestimated the prevalence of appendicitis with 6%. The negative predictive value of appendicitis was 94.0% and the accuracy 91.3%†	Andersson [60]	8298378	1994	Histological examination of excised tissue (in patients undergoing appendectomy)
Inguinal hernia	17/18 (94)	Only primary diagnoses were evaluated. NPV = 882/882 (100)	Nilsson [7]	8114596	1994	MR
**Other disorders**						
Achalasia	67/83 (81)		Zendehdel [61]	17488250	2007	MR
Prostate hyperplasia	14/14 (100)	Only primary diagnoses were evaluated. NPV = 882/886 (99.5)	Nilsson [7]	8114596	1994	MR
Asthma	14/15 (93)	Only primary diagnoses were evaluated. NPV = 885/885 (100)	Nilsson [7]	8114596	1994	MR
Abdominal pain (observation for abdominal pain)	33/37 (89)	Only primary diagnoses were evaluated. NPV = 860/863 (99.7)	Nilsson [7]	8114596	1994	MR
Preeclampsia	137/148 (92.6)		Ros [39]	9620050	1998	MR
Endometriosis	Without ovarial cancer: 615/628 (97.9% correct). With ovarial cancer: 220/225 (97.8% correct)		Melin	-	(PC, Feb 3)	MR
Foot ulcer (only)	249/250 (99.6) admissions to hospital were correct with regards to ICD-coding	Based on 117 patients with deep foot infections and type 1 or 2 diabetes, referred to multidisciplinary foot-care team. Specificity for foot ulcers and concomitant diabetes was 98%.	Ragnarson-Tennvall [46]	11123504	2000	MR
Connective tissue disease (CTD)	71/91 (78%)	In this study CTD included rheumatoid arthritis, systemic lupus erythemathosus, sclerodermia, Sjögren's syndrome, dermatomyositis, polymyositis etc.	Nyren [62]	9492663		MR
Acute pancreatitis	695/602 (98.8)	Among 602 patients with a primary or secondary diagnosis of acute pancreatitis in the IPR. 84.0% had a definitive acute pancreatitis and another 14.8% a probable acute pancreatitis.	Lindblad		(PC, Feb 6)	MR + laboratory tests and radiological imaging

PC, Personal communication: All personal communications took place in 2010 (exact date is listed in the table).

CDT, Connective tissue disease. GBS, Guillain-Barré Syndrome; HF, Heart Failure, IBD, Inflammatory bowel disease. IPR, Inpatient Register.

MR, compared with Medical Records (patient charts).

PPV, Positive Predictive Value.

NPV, Negative Predictive Value.

ULSAM, Uppsala Longitudinal Study of Adult Men) cohort.

*From ICD-7 through ICD-9, no distinction was made between type 1 and type 2 diabetes. For practical reasons "diabetes" has been listed as an autoimmune disorder.

† These studies took place when the county in question did not yet report inpatient data to the IPR, but results are deemed valid for the IPR.

**Table 4:** Validation of diagnoses in the Swedish Inpatient Register by sensitivity

Diagnosis	Proportion identified through the IPR (%) (sensitivity)	Comment	Main Author	PubMed ID	Year	Gold Standard (Reference)
**Cardiovascular disease**						
Myocardial Infarction (MI)	54/59 (91.5)	Cross-sectional, 1-year retrospective study. ≥20 year-olds from Degerfors area.	Elo [14]	19084244	2009	Data were obtained from IPR, hospital-based outpatient care and primary health care
Myocardial infarction	99/128 (77)		Merlo [63]	10870938	2000	Men born 1914
Myocardial infarction	113/144 (79)		Merlo [63]	10870938	2000	The Skaraborg Hypertension Study
Myocardial Infarction	3201/4148 (77.2)	The researchers identified all MIs in Stockholm county in 1973 through the local IPR and the national Cause of Death Register (restricted to individuals living in Stockholm).	Ahlbom [64]	721364	1978	Swedish Cause of Death Register
Myocardial infarction	4746/5832 (81.4) of MI cases in community registers were found in IPR or the Cause of Death Register.	IPR and Cause of Death Register data from 1972-1981 for regions with IPR registers at the time. 81% of cases in community registers were found in IPR or Cause of Death Register. Meanwhile 85% of cases in IPR and the Cause of Death Register were found in Community registers (disregarding non-matching fatal cases).	Hammar [65]	2066207	1991	Community registers with myocardial infarctions
Angina Pectoris	86/196 (43.9)	Cross-sectional, 1-year retrospective study. ≥20 year-olds from Degerfors area.	Elo [14]	19084244	2009	Data were obtained from IPR, hospital-based outpatient care and primary health care
Acute coronary syndrome (MI or unstable angina pectoris)	IPR from emergency department missed 2% of 218 patients with acute coronary syndrome.	Discharge diagnoses from emergency department, Lund University Hospital.	Forberg [66]	18804783	2009	MR. The study evaluates different methods to identify acute coronary syndrome in patients, using information not available in the IPR (e.g. ECG measurements).
Non-specified ischemic heart disease	44/206 (21.4)	Cross-sectional, 1-year retrospective study. ≥20 year-olds from Degerfors area.	Elo [14]	19084244	2009	Data were obtained from IPR, hospital-based outpatient care and primary health care
Stroke (non-subarachnoidal hemorrhage)	318/377 (84.4)	Data from a local stroke incidence study at the Örebro University Hospital.	Appelros	-	(PC, Feb 22)	Local stroke incidence study
Non-fatal strokes	3492/3732 (93.6) of all cases 3492/3562 (98.0) of estimated hospital cases	Patients aged 25-74 years in 1985-89. Criteria for acute stroke from MONICA study were comparable to WHO criteria.	Stegmayr [38]	1291884	1992	MONICA-population based stroke register
Stroke	75/79 (95)		Merlo [63]	10870938	2000	The Skaraborg Hypertension Study
Stroke	76/81 (94)		Merlo [63]	10870938	2000	"Men born 1914"
Stroke	384/456 (84.2)	First-time stroke. Restricted to the Lund county.	Hallström [67]	17156265	2007	*
Stroke/Transient ischemic A.	217/232 (93.5)	ULSAM.	Wiberg	-	(PC, Feb 12)	ULSAM
Hypertension	5,886/42,796 (13.7)	IPR data from the county Östergötland.	Wiréhn [68]	17786807	2007	County registers of primary health care, outpatient hospital care and inpatient care
Hypertension	74/838 (8.8)	Cross-sectional, 1-year retrospective study. ≥20 year-olds from Degerfors area.	Elo [14]	19084244	2009	Data were obtained from IPR, hospital-based outpatient care and primary health care
Gestational hypertension	108/166 (65.1)		Zetterström	-	(PC, March 3)	All pregnant women in Sweden have their blood pressure examined.
Lipid disorders	19/186 (10.2)	Cross-sectional, 1-year retrospective study. ≥20 year-olds from Degerfors area.	Elo [14]	19084244	2009	Data were obtained from IPR, hospital-based outpatient care and primary health care
**Autoimmune/immune-mediated diseases**						
Rheumatoid arthritis (RA)	489/1150 (42.5)	Malmö, Sweden, 1990-1994. According to the RA criteria of the American College of Rheumatology [41].	Turesson [42]	10461483	1999	Data from all rheumatologists and general practictioners in Malmö city, 1997
Diabetes, type 1 and 2*	92/394 (23.3)	Cross-sectional, 1-year retrospective study. ≥20 year-olds from Degerfors area.	Elo [14]	19084244	2009	Data were obtained from IPR, hospital-based outpatient care and primary health care
Type 1 and type 2 diabetes mellitus *	349/436 (80.0) patients with diabetes in "source 1" could be identified in the IPR	The researchers evaluated the presence of type 1 or type 2 diabetes among patients with atrial fibrillations at the Södersjukhuset Hospital. All but 8 patients in this validation had type 2 diabetes.	Friberg	-	(PC, Feb 8)	MR + biochemistry data + data on insulin or other medication against diabetes
Type 1 and type 2 diabetes mellitus*	4,348/18,134 (24.0)	IPR data from the county Östergötland.	Wiréhn [68]	17786807	2007	County registers of primary health care, outpatient hospital care and inpatient care
Both type 1/2 diabetes and foot ulcer	235/280 (83.9) admissions could be identified through the IPR	Based on 117 patients with deep foot infections and type 1 or 2 diabetes, referred to multidisciplinary foot-care team. Specificity for foot ulcers and concomitant diabetes was 98%.	Ragnarson- Tennvall [46]	11123504	2000	MR
**Infectious disease**						
CNS infection in intensive care	ICD-9: 21/22 (95.4) ICD-10: 21/28 (75.0)	Specificity was 99.6% (ICD-9) and 99.7% (ICD-10). IPR data obtained through both primary and secondary diagnoses.	Gedeborg [69]	17208121	2007	ICU database diagnoses as reference
Pneumonia in intensive care	ICD-9: 89/185 (48.1) ICD-10: 116/221 (52.5)	Specificity was 95.9% (ICD-9) and 95.8% (ICD-10). IPR data obtained through both main and secondary diagnoses. Using only primary diagnoses for pneumonia the specificity increased slightly but the sensitivity decreased to 31.2% and 38.2% respectively.	Gedeborg [69]	17208121	2007	ICU database diagnoses as reference
Sepsis in intensive care	ICD-9: 85/186 (45.7) ICD-10: 94/179 (52.5)	Specificity was 97.5% (ICD-9) and 92.6% (ICD-10). IPR data obtained through both main and secondary diagnoses.	Gedeborg [69]	17208121	2007	ICU database diagnoses as reference, with inclusion in sepsis trials (IST) as secondary reference
Tuberculosis	12/15 (80)	Evaluation in patients with concomitant rheumatoid arthritis	Askling [70]	15986370	2005	MR
**Other disorders**						
Carotid, infrainguinal bypass and aortic aneurysm (AAA) procedures	10,861/11,638 (93.3) of patients identified through Swedvasc (Svenska kärlregistret) or the IPR could be identified in the IPR	(Swedvasc) started in 1987 and became nationwide 1994. In the paper by Troëng et al data on infraingunal and carotid bypass originated from 5 years (2000-2004), while data on aortic aneurysms originated from 1 year (2006). Divided the sensitivity figures were: Carotid 96.8%; Infrainguinal 92.3% and aortic aneurysm 88.9%.	Troëng [15] And by	18851920	2008	The Swedish Vascular Register (Swedvasc)
Asthma	1,377/18,451 (7.5)	IPR data from the county Östergötland.	Wiréhn [68]	17786807	2007	County registers of primary health care, outpatient hospital care and inpatient care
Dementia	23/87 (26)	Participants originated from the study "Aging in women and men: a longitudinal study of gender differences in health behaviour and health among the elderly (as part of the Swedish Twin Register). The specificity for dementia was 97% (399/411)	Dahl [71]	18007116		MR + memory test scores etc.
Dementia	41.3% for prevalent cases and 42.3% for incident cases.	Participants originated from the study "Aging in women and men: a longitudinal study of gender differences in health behaviour and health among the elderly (as part of the Swedish Twin Register). The specificity for dementia was 97% (399/411)	Rizutto		(PC, May 14)	Comparison between cases detected in IPR with cases derived from the Kungsholmen (KP) and SNAC-K Projects [72]
Foot ulcer (only)	249/280 (88.9%) of admissions could be identified through the IPR	Based on 117 patients with deep foot infections and type 1 or 2 diabetes, referred to multidisciplinary foot-care team.	Ragnarson- Tennvall [46]	11123504	2000	MR
Chronic obstructive pulmonary disease	1,290/4,812 (26.8)	IPR data from the county Östergötland.	Wiréhn [68]	17786807	2007	County registers of primary health care, outpatient hospital care and inpatient care

PC, Personal communication: All personal communications took place in 2010 (exact date is listed in the table).

ICU, Intensive Care Unit. MI, Myocardial Infarction. MR, compared with Medical Records (patient charts). RA, Rheumatoid arthritis.

ULSAM, Uppsala Longitudinal Study of Adult Men) cohort.

*Data from all departments at the Lund Hospital including the emergency unit, general practitioners, community nurses, physicians at the Neurology department, autopsy registers, Forensic department, computerized searches of outpatient medical records

## Results

With few exceptions, validation of ICD codes from the IPR was made by comparing registered diagnoses in the IPR with information in medical records (Tables 3 and 4). The positive predictive values (PPVs) of IPR diagnoses were 85-95% for most diagnoses (3-digit level, see Table 3). In a review of patients dying in hospital 90-98% of patients with a primary discharge diagnosis of malignancy had the same malignancy as the underlying cause of death [5]. In addition, 90.3% of those with a primary discharge diagnosis of myocardial infarction (MI) had MI as the underlying cause of death and with a similar proportion of those with other vascular diseases (89.0%). Agreement between discharge diagnosis and death certificate was slightly lower for traffic accidents (87.8%), meningitis (74.3%) and ulcer of the stomach or duodenum (69.9%) to name a few [5].

Sensitivity of the IPR was high (above 90%) for MI [14] as well as for surgery for carotid stenosis, surgery on the carotid arteries, or surgery on the arteries in the leg (infrainguinal) and aorta [15](Table 4) but low for lipid disorders and hypertension [14]. Few studies have examined to what extent an individual without a specific disease is assigned an ICD code for that disease.

Some hospital admissions are due to trauma and not disease. In 2008, Backe et al [16] used ambulance records as gold standard to examine the proportion of injuries and suffocations that were then recorded in the IPR. Agreement between the two data sources varied, with high agreement for "falls" (W00-W19; 93.9%) but lower for "road traffic accidents" (ICD-10: V01-V99) and "suffocation, drowning/near drowning, etc." (ICD-10: W64-85), where the IPR recorded less than 50% of all injuries noted in the ambulance reports.

Several studies have examined date of hospital admission. For instance, Nordgren found that for 62% (257/413) of spinal cord injuries, the hospital admission date agreed with the injury date (≤2 days within the injury date [17]).

## Discussion

This review found a high PPV for the majority of evaluated diagnoses but a lower sensitivity. The PPVs reported in this review are similar to those in the Danish IPR (febrile seizures in children: 93%[18], MIs: 92-94%[19], venous thromboembolism: 75%[20]). Furthermore, US hospital data suggest a PPV of about 90% for some diagnoses (e.g., acromegaly: 76% of the patients had a definite diagnosis and 14% a probable diagnosis [21]).

The proportion of valid diagnoses in the IPR is probably higher in patients with severe as opposed to mild disease and higher among patients with causally related complications in contrast to those without complications. Baecklund et al reported that the IPR diagnosis of rheumatoid arthritis was correct in 93.5% of individuals with later lymphoma but only in 87.1% in individuals who had not developed later lymphoma [22]. In this case the positive association between lymphoma and rheumatoid arthritis leads to higher specificity for rheumatoid arthritis in patients with lymphoma.

There are several ways to increase the specificity and the PPV of a diagnosis in the IPR. In a paper on sepsis in celiac disease by Ludvigsson et al [23] sensitivity analyses were performed among patients with (1) sepsis diagnosed in a department of infectious diseases (i.e. in a department where sepsis is likely to be correctly diagnosed), (2) sepsis listed as the *primary *diagnosis and (3) the risk of having at least two hospital admissions with sepsis. All these measures could increase the specificity of a diagnosis. For instance, there is a risk that individuals discharged from a dermatology department with a diagnosis of MI (ICD-10: I20.9) actually had an incorrectly recorded eczema (ICD-10: L20.9). When Parikh et al examined parity and risk of later cardiovascular disease, they restricted their discharges to patients with a primary diagnosis of cardiovascular disease (or death from cardiovascular disease)[24]. In their recent paper on schizophrenia, substance abuse and violent crime Fazel et al resolved to study patients with at least two hospital admissions with schizophrenia [25].

The extent to which a condition has been reported and recorded in the IPR depends on several factors [26], including care-seeking behaviour of an individual, access to health care and the propensity of a physician to admit a patient. Hospital fees, however, are no major obstacle to inpatient care access in that the (public) health system in Sweden is almost free of charge.

Over time, an increasing number of patients are treated as outpatients [27], a trend largely driven by economic restraints but also by data indicating that the prognosis of some diseases (e.g., stroke) has an improved prognosis in ambulatory care [28]. The trend towards outpatient care suggests that the sensitivity of the IPR may have decreased in recent years for some diseases. In fact, our validation showed that the IPR has low sensitivity for hypertension and lipid disorders. The introduction of day care anaesthesia has resulted in that certain procedures, such as small-intestinal biopsy preceding a diagnosis of celiac disease [29], which previously required inpatient care, are nowadays often performed on an outpatient basis.

When Elmberg et al estimated mortality in patients with hereditary haemochromatosis (HH)[30], they found a relative risk of death of 2.15 among HH patients identified through the IPR, but only 1.09 in patients identified through regional clinic registers and 1.15 in those identified through outpatient data sources [30]. Some evidence suggests that patients with a certain disorder identified through the IPR may suffer from more intense disease than the average patient and be at higher risk of complications than patients identified outside the IPR (a phenomenon sometimes called Berkson's bias [31]).

Another issue that deserves attention is that the first recorded admission with a disorder is not always equal to the *incident *admission. According to patient chart reviews, 1 in 3 patients with a hospital admission for stroke had had an earlier stroke (L. Olai, personal communication, Feb 4, 2010). In an effort to separate incident admissions from readmissions some authors have suggested using prediction models combining information from current and previous records in the IPR [32]. It should be noted that the Swedish ICD system does contain a number of codes representing late effects of disease, such as ICD code I69 ("late effects of cerebrovascular disease").

A number of non-medical factors influence the coding of hospital discharges. Although originally used to collect data on health care use, today the IPR coding is also used as the basis for management and financing. Some hospitals have introduced compulsory use of certain secondary codes (when such codes apply) because these codes generate extra funding (e.g., a secondary code of diabetes mellitus is "valuable"). Further, international research suggests that the coding pattern may differ between hospitals and general practice [33]. Financial incitements have therefore led to a "diagnostic drift" in which more secondary diagnoses are listed [27] and where it is financially more rewarding to assign a patient a severe primary diagnosis than a severe secondary diagnosis (e.g., type 1 diabetes is more "valuable" as a primary diagnosis than as a secondary diagnosis). The effects of financial incitements on ICD coding have probably been underestimated and are likely to have changed the epidemiological pattern. A standardized behaviour of assigning ICD codes is therefore of importance for all stakeholders, including the Swedish state [27].

Despite the extensive scope of the IPR, there is still a need for additional variables (Additional file 3), including laterality, index admission, earlier comorbidity and risk factors (e.g., smoking).

## Conclusion

In conclusion, the Swedish IPR is a valuable resource for large-scale register-based research. A number of diagnoses have already been validated by the NBHW and by individual researchers. Current data suggest that the overall PPV of diagnoses in the register is about 85-95%.

## Competing interests

The authors declare that they have no competing interests.

The funding organizations played no role in the design or conduct of the study, in the collection, management, analysis, or interpretation of the data, and did not participate in preparation, review, or approval of the manuscript.

## Authors' contributions

JFL designed the study, collected the data, and wrote the first draft of the paper. JFL, EA, AE, MF, JLK, CR and POO examined the papers that were used when examining the validity of the Swedish Inpatient Register. All authors contributed to the interpretation of the results. All authors read and approved the final manuscript.

## List of abbreviations

IPR: Swedish Inpatient Register (Slutenvårdsregistret); MI: Myocardial infarction; NBHW: National Board of Health and Welfare (Socialstyrelsen); PIN: Personal Identity Number.

## Pre-publication history

The pre-publication history for this paper can be accessed here:

http://www.biomedcentral.com/1471-2458/11/450/prepub

## Supplementary Material

Additional file 1 **Detailed description of the laws and regulations governing the Swedish Inpatient Register**. Please see Title. Click here for file

Additional file 2 **Population data used to construct Figures 2 and 3**. Population of Swedish Counties in1960 and 1990. Click here for file

Additional file 3 **Variables that could potentially add value to the Inpatient Register**. This file lists a number of variables that could be added to the Inpatient Register. Click here for file
