# Supplementary material for: External review and validation of the Swedish national inpatient register
Source: BMC Public Health. 2011 Jun 9;11:450. doi: 10.1186/1471-2458-11-450 (PMC3142234; doi:10.1186/1471-2458-11-450)
Supplement: Additional file 1 — Detailed description of the laws and regulations governing the Swedish Inpatient Register. Please see Title. [file 1471-2458-11-450-S1.DOC]

# Additional files

**Additional file 1.**

**Title: Detailed description of the laws and regulations governing the Swedish Inpatient Register.**

The IPR is regulated by the Health Care Data Register Act (1998:543; Lag om hälsodataregister) and the IPR ordinance (2001:707; Förordning om patientregister hos Socialstyrelsen). It is mandatory for all physicians, private and publicly funded, to deliver data to the IPR. Data from the IPR are subjugated to the Health and Medical Services Act (1982:763; Hälso och sjukvårdslag) and the Patient Data Act (2008:355; Patientdatalag). Of special importance to the regulation of Swedish medical research and health care is also the Public Access to Information and Secrecy Act (2009:400, Offentlighets- och sekretesslagen).
