# Supplementary material for: External review and validation of the Swedish national inpatient register
Source: BMC Public Health. 2011 Jun 9;11:450. doi: 10.1186/1471-2458-11-450 (PMC3142234; doi:10.1186/1471-2458-11-450)
Supplement: Additional file 2 — Population data used to construct Figures 2 and 3. Population of Swedish Counties in1960 and 1990. [file 1471-2458-11-450-S2.DOC]

# Additional files

**Additional file 2**

**Title: Population data used to construct Figures 2 and 3.**

**Description: Population of Swedish Counties in1960 and 1990.**

| **County** | **Proportion of National Population *** | **1960** | **1990** |
| --- | --- | --- | --- |
| 01 Stockholm | 0,1803 | 1271014 | 1641669 |
| 03 Uppsala | 0,0268 | 167722 | 268835 |
| 04 Södermanland | 0,0301 | 227807 | 255636 |
| 05 Östergötland | 0,0473 | 357601 | 403011 |
| 06 Jönköping | 0,0370 | 285348 | 308290 |
| 07 Kronoberg | 0,0209 | 158867 | 177882 |
| 08 Kalmar | 0,0297 | 235612 | 241102 |
| 09 Gotland | 0,0069 | 54196 | 57108 |
| 10 Blekinge | 0,0184 | 144466 | 150564 |
| 11 Kristianstad | 0,0339 | 256395 | 289278 |
| 12 Malmöhus | 0,0871 | 626086 | 779309 |
| 13 Halland | 0,0262 | 169995 | 254725 |
| 14 Göteborg och Bohus | 0,0848 | 625670 | 739945 |
| 15 Älvsborg | 0,0507 | 374683 | 441391 |
| 16 Skaraborg | 0,0328 | 249948 | 276830 |
| 17 Värmland | 0,0359 | 291074 | 283110 |
| 18 Örebro | 0,0334 | 262321 | 272513 |
| 19 Västmanland | 0,0306 | 232973 | 258487 |
| 20 Kopparberg | 0,0359 | 286047 | 289067 |
| 21 Gävleborg | 0,0364 | 293246 | 289294 |
| 22 Västernorrland | 0,0343 | 285676 | 261155 |
| 23 Jämtland | 0,0172 | 139799 | 135726 |
| 24 Västerbotten | 0,0306 | 239619 | 251968 |
| 25 Norrbotten | 0,0328 | 261802 | 263735 |

*Average between 1960-1990.

Please note, since 1990 some of these counties have merged.
