# Supplementary material for: External review and validation of the Swedish national inpatient register
Source: BMC Public Health. 2011 Jun 9;11:450. doi: 10.1186/1471-2458-11-450 (PMC3142234; doi:10.1186/1471-2458-11-450)
Supplement: Additional file 3 — Variables that could potentially add value to the Inpatient Register. This file lists a number of variables that could be added to the Inpatient Register. [file 1471-2458-11-450-S3.DOC]

# Additional files

**Additional file 3. Variables that could potentially add value to the Inpatient Register.**

**Description: This file lists a number of variables that could be added to the Inpatient Register.**

| Laterality | Currently it is not recorded if an amputation is carried out on the right or left side. |
| --- | --- |
| Index admission (actually discharge) | At present there is no variable in the IPR indicating if an admission is the first for a certain disorder. |
| ICD classification | Since 1964, Sweden has used four ICD classifications. The transition has differed between counties. A variable indicating the ICD classification used for each hospital admission would be desirable. |
| Earlier comorbidity or “Present on admission” (POA) | A variable indicating earlier comorbidity could help researchers with respect to the potential confounding of comorbidity. Furthermore, it may differentiate between diagnoses present on admission and what diagnoses occurred during hospital admission (and constituted a complication). For instance, a patient with a primary diagnosis of hip fracture and a secondary diagnosis of venous thromboembolism may have had the thromboembolism on admission or had it after admission. |
| Other risk factors. | The IPR does not contain any data on risk factors, most prominent perhaps being smoking. Although smoking data are available in subsets of the Swedish population (pregnant women after 1982[21]; subsets of young men in the Swedish Conscription Register and in certain research cohorts), there are no smoking data in the IPR. Nor are there any data on use of alcohol and body mass index (BMI). |

None of the above variables are currently listed in the Inpatient Register.
